# The Theoretical Foundations of ECHO: The Key to Interprofessional Innovation in Geriatrics Education

**DOI:** 10.1093/geroni/igaf122.747

**Published:** 2025-12-31

**Authors:** Phillip Clark

**Affiliations:** University of Rhode Island, Kingston, Rhode Island, United States

## Abstract

“There is nothing so practical as a good theory,” observed social psychologist Kurt Lewin. In developing innovative methods of interprofessional geriatrics education, this maxim applies to how the theoretical foundations of Project ECHO can enhance educators’ ability to apply its dual concepts of “tele-mentoring” and “tele-networking” to improve geriatric care. The multiple, complex, and chronic conditions of many older adults require interprofessional and interorganizational teamwork to provide Age-Friendly care. Two of the educational theories underpinning ECHO are especially relevant to addressing this need: (1) community of practice, and (2) single- and double-loop learning. Community of practice requires an intentional commitment of professionals to an ongoing group for learning and improving care. In ECHO, it provides the structure within which educational methods are employed and supports members in coping with challenging patients and situations. The ECHO principle of “all teach, all learn” is the foundation of this community. Single-loop learning involves acquiring professional knowledge to improve care and is embodied in didactic presentations. In contrast, double-loop learning entails transforming familiar modes of practice by recognizing the limits of one’s own discipline and supporting interprofessional collaboration for Age Friendly care. This outcome is based on the ECHO case presentation and discussion. Future innovations incorporating these theoretical principles are presented: (1) advantage of learning 4M care within a community of practice, (2) need for interprofessional teamwork made explicit in ECHO programs, and (3) requirement for mixed-methods evaluation of ECHOs to capture the impacts of changes in practice on patient care and outcomes.

